# Conservative management of a delayed enterocutaneous fistula following emergency distal gastrectomy using sequential somatostatin analogues

**DOI:** 10.1093/jscr/rjag176

**Published:** 2026-03-16

**Authors:** Alexandra Zalums, Angus Hibberd

**Affiliations:** The University of Sydney School of Rural Health - Orange Campus, 1530 Forest Road, Orange, NSW 2800, Australia; Department of General Surgery, Orange Health Service, 1530 Forest Road, Orange, NSW 2800, Australia

**Keywords:** gastric ulcer perforation, enterocutaneous fistula, somatostatin analogue

## Abstract

Enterocutaneous fistulas (ECFs) are serious postoperative complications associated with significant morbidity and mortality. Somatostatin analogues are widely used to reduce fistula output, although their effect on definitive closure remains uncertain. Evidence regarding long-acting formulations in postoperative ECFs is limited, particularly in the context of outpatient management. We report a case of delayed postoperative ECF following emergency distal gastrectomy for perforated gastric ulcer. The patient was managed non-operatively using sequential administration of short-acting Octreotide followed by long-acting Lanreotide. Conservative management avoided reoperation despite recurrent collections and prolonged fistula activity. Octreotide therapy was associated with reduction in fistula output, and transition to monthly Lanreotide enabled simplified outpatient care. The fistula subsequently closed without surgical re-intervention; however, spontaneous closure cannot be excluded as a contributing factor. This case emphasizes surgical decision-making in a hostile postoperative abdomen and the sequential use of somatostatin analogues in conservative community-based care.

## Introduction

Enterocutaneous fistulas (ECFs) remain among the most challenging complications following abdominal surgery. Up to 85% arise postoperatively, with reported mortality rates of 5%–21%, most commonly related to sepsis, malnutrition, and electrolyte imbalance [1, 2]. High-output fistulas are associated with prolonged hospitalization, skin injury, and substantial patient distress [3].

Standard management prioritizes sepsis control, fluid and electrolyte optimization, nutritional support, and meticulous wound care [1, 4]. Pharmacologic reduction of gastrointestinal secretions using somatostatin or its analogues is widely practised, with evidence supporting reductions in fistula output and time to closure, though not consistently improving spontaneous closure rates or mortality [5]. Long-acting formulations such as Lanreotide offer potential advantages for outpatient management, but data remain limited [3, 6, 7].

We describe a complex case of delayed postoperative ECF following emergency distal gastrectomy, highlighting surgical decision-making and the practical role of sequential somatostatin analogue therapy.

## Case report

A woman in her 60s presented to a regional hospital in New South Wales, Australia, with acute upper abdominal pain and syncope. Her medical history included hypertension, hypothyroidism, hypercholesterolaemia, gastroesophageal reflux disease, and a 30 pack-year smoking history. Examination revealed generalized peritonism, and laboratory testing demonstrated acute kidney injury.

CT imaging confirmed a perforated distal gastric ulcer with free intraperitoneal air and fluid, as well as pancreatic calcification consistent with chronic pancreatitis. After resuscitation, she underwent emergency laparoscopic conversion to open distal gastrectomy with duodenal exclusion and gastrojejunostomy. Intraoperatively, extensive contamination and a large posterior gastric perforation were encountered. The duodenum was inflamed with distorted tissue planes. The duodenal stump was closed primarily; tube duodenostomy was considered but deferred due to apparent security of closure and haemodynamic stability. A subhepatic drain was placed.

**Figure 1:**
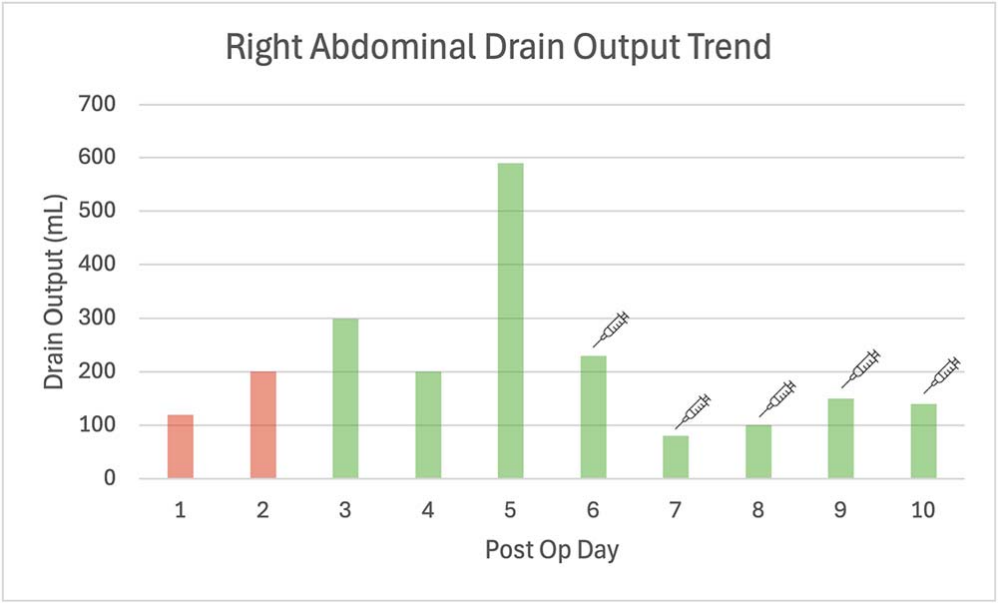
Bar graph of post operative daily drain outputs, where Days 1 and 2 the drain fluid quality was haemoserous, becoming bilious from Day 3 onwards. The syringe symbol indicates the introduction of Octreotide 100 μg subcutaneous three times daily injection.

On postoperative Day (POD) 2, bilious drain output developed, peaking at 590 ml/day by POD 5. As the patient remained clinically stable without peritonism, reoperation was avoided. Octreotide 100 μg subcutaneously three times daily was commenced, resulting in gradual reduction in output (Fig. 1). She was discharged on POD 10 with community nursing support.

**Figure 2:**
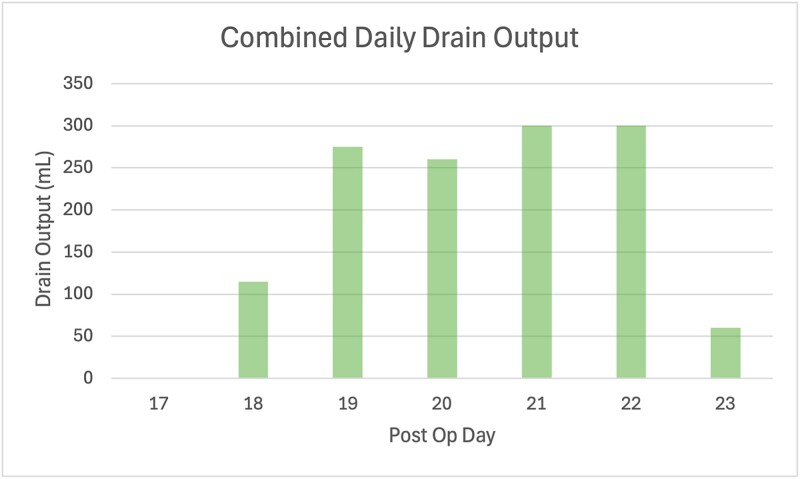
Bar graph of combined daily drain output volumes following wound washout and revision, where the volume is a tally of the right abdominal drain and two subcutaneous wound drains. The quality of the drain fluid was bilious.

On POD 15, she re-presented with sudden bilious discharge from the upper laparotomy wound due to drain obstruction. CT imaging demonstrated a complex anterior abdominal wall collection, and CT cholangiography confirmed a leak from the duodenal stump tracking to the wound, consistent with an ECF. Gastroscopy showed an intact gastrojejunal anastomosis. She underwent wound washout, fascial reinforcement, and placement of subcutaneous drains. Bilious output recurred but gradually decreased over subsequent weeks, allowing eventual drain removal (Fig. 2).

**Figure 3:**
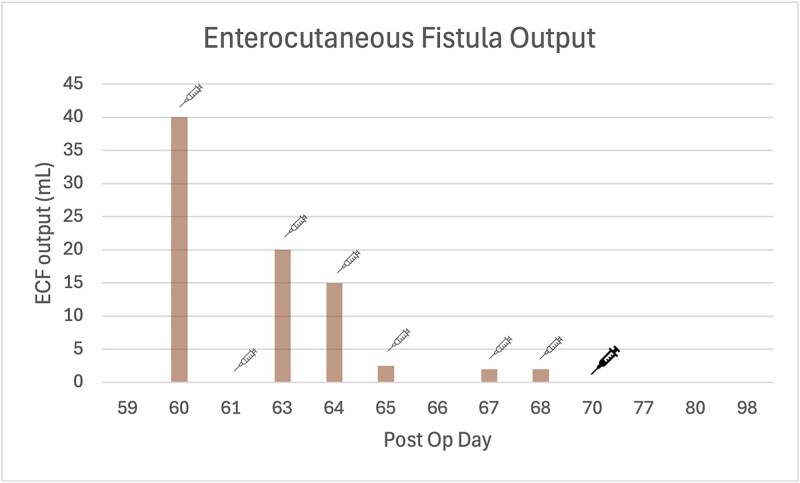
Bar graph of ECF output measurements, where Octreotide 100 μg subcutaneous three times daily injection was re-introduced post op Day 60 (as indicated by fine line syringe icon), and was switched to Lanreotide 60 mg subcutaneous injection post op Day 70 (as indicated by bold line syringe icon). The quality of the ECF fluid was enteric.

Two months after the index operation, she re-presented with a recurrent ventral abdominal wall collection leaking purulent fluid. Imaging and fluid amylase levels confirmed ongoing enteric communication. She was treated with intravenous antibiotics, fluid resuscitation, proton pump inhibition, and recommenced on Octreotide.

Following discharge, she continued Octreotide 100 μg subcutaneous injections three times daily then transitioned to monthly Lanreotide 60 mg subcutaneous injections to facilitate outpatient care. Fistula output continued to decrease and subsequently ceased (Fig. 3). While temporal association was observed, spontaneous fistula closure remains a plausible alternative explanation.

## Discussion

This case illustrates the complex and often unpredictable natural history of postoperative ECFs following upper gastrointestinal surgery. The delayed externalization of a duodenal stump leak through a superficial wound sinus, rather than immediate bowel-to-skin communication, is uncommon and likely reflects multifactorial influences including impaired tissue healing and complex fistula anatomy [1].

Chronic pancreatitis and continued smoking likely contributed both to operative difficulty and delayed fistula formation. Although tube duodenostomy may reduce the risk of uncontrolled bile leakage in selected high-risk duodenal closures, its omission in this case was reasonable given intraoperative findings and haemodynamic stability [8].

A central learning point is surgical decision-making in a hostile postoperative abdomen. Reoperation in the setting of recent upper gastrointestinal surgery carries substantial morbidity due to the presence of inflammation, contamination, and distorted anatomy [9]. In this patient, clinical stability permitted a conservative strategy focused on sepsis control, nutritional optimization, and meticulous wound care.

Somatostatin analogues formed part of a broader conservative management strategy aimed primarily at reducing fistula output and facilitating supportive cares. Consistent with existing meta-analyses, these agents are best understood as adjuncts that may shorten time to functional quiescence rather than definitively determining fistula closure [1, 6]. Importantly, spontaneous closure remains a well-recognized outcome in appropriately managed postoperative fistulas and cannot be excluded as a contributing factor in this case [7].

A practical aspect of management was the transition from short-acting Octreotide to long-acting Lanreotide. Frequent subcutaneous injections impose significant burden on patients and community healthcare services. Switching to a long-acting formulation enabled simplified outpatient treatment and improved feasibility of community-based care—an outcome seldom evaluated in existing trials [7].

Overall, this case underscores that successful management of postoperative ECF depends less on any single pharmacological intervention and more on careful patient selection, vigilant monitoring, and judicious surgical restraint. Sequential use of short- and long-acting somatostatin analogues may serve as a practical adjunct within a comprehensive conservative management framework, especially when reoperation carries high risk. Further prospective studies are needed to better define the role of long-acting somatostatin analogues in facilitating outpatient ECF care.
